# The high comorbidity burden of the hepatitis C virus infected population in the United States

**DOI:** 10.1186/1471-2334-12-86

**Published:** 2012-04-11

**Authors:** Karly S Louie, Samantha St Laurent, Ulla M Forssen, Linda M Mundy, Jeanne M Pimenta

**Affiliations:** 1 Worldwide Epidemiology, GlaxoSmithKline, 1-3 Ironbridge Road, Stockley Park, Uxbridge UB11 1BT, UK; 2 Worldwide Epidemiology, GlaxoSmithKline, Waltham, MA, USA; 3 Worldwide Epidemiology, GlaxoSmithKline, 1250 South Collegeville Road, PO Box 5089, Collegeville, PA 19426-0989, USA

## Abstract

**Background:**

Chronic hepatitis C (HCV) disease can be complicated with comorbid conditions that may impact treatment eligibility and outcomes. The aim of the study was to systematically review comorbidities and symptoms in an HCV infected population, specifically assessing comorbidities associated with HCV anti-viral treatment and disease, as well as comparing comorbidities between an HCV infected and uninfected control population.

**Methods:**

This was a retrospective cohort study within a United States medical claims database among patients with chronic HCV designed to estimate the two-year period prevalence of comorbidities. Patients with two HCV diagnosis codes, 24 months of continuous health insurance coverage, and full medical and pharmacy benefits were included.

**Results:**

Among a chronic HCV cohort of 7411 patients, at least one comorbid condition was seen in almost all patients (> 99%) during the study period. HCV-infected patients reported almost double the number of comorbidities compared to uninfected controls. Of the 25 most common comorbidities, the majority of the comorbidities (n = 22) were known to be associated with either HCV antiviral treatment or disease. The five most frequent comorbidities were liver disease [other] (37.5%), connective tissue disease (37.5%), abdominal pain (36.1%), upper respiratory infections (35.6%), and lower respiratory disease (33.7%). Three notable comorbidities not known to be associated with antiviral treatment or disease were benign neoplasms (24.3%), genitourinary symptoms & ill-defined conditions (14.8%), and viral infections (13.8%).

**Conclusions:**

This US medically insured HCV population is highly comorbid. Effective strategies to manage these comorbidities are necessary to allow wider access to HCV treatment and reduce the future burden of HCV disease and its manifestations.

## Background

Hepatitis C virus (HCV) is the etiology of the most common chronic bloodborne infection in the United States (US), affecting at least 4.1 million people, of whom 3.2 million are chronically infected [1]. Chronic infection can lead to progressive development of liver fibrosis to cirrhosis, end-stage liver disease, and hepatocellular carcinoma (HCC) in 5-20% of patients within the first two decades of initial infection [2]. Complications of chronic HCV are projected to increase dramatically over the next few decades [3], placing an ever greater burden on health systems of a disease where the annual health care costs are already estimated at over one billion dollars [4]. Known co-factors for the progression of liver disease include duration of infection, male gender, high-levels of alcohol consumption, hepatitis B virus (HBV) infection, and human immunodeficiency virus (HIV) infection. Other data show that metabolic factors such as steatosis, being overweight or obese may also contribute to progression [5]. Several of these factors are comorbid conditions that may also impact HCV treatment eligibility and outcomes. Other conditions that are known contraindications for HCV treatment are severe depressive disorders, hyperthyroidism, hypertension, heart failure, coronary heart disease, diabetes, and pulmonary disease [2].

The current standard-of-care for chronic HCV treatment is combination pegylated-interferon and ribavirin with the addition of boceprevir or teleprevir for genotype 1 patients only [6]. Among patients on pegylated-interferon and ribavirin only, treatment is only efficacious in approximately 55% of treatment-naïve patients [7]. About 75% of treated patients experience one or more side effects requiring discontinuation or dose modification of treatment [8]. Specifically, antiviral treatment may exacerbate certain conditions such as those with anaemia, thrombocytopenia or leukopenia, which can further complicate treatment outcomes by increasing the number and severity of side-effects [9]. Consequently, many patients are excluded from treatment, or have treatment deferred or compromised because of their comorbid conditions.

To maximize the benefit of current and future HCV therapeutic regimens, a greater understanding of the most frequent comorbidities associated with HCV is needed. Successfully managing these comorbidities may lead to increased treatment eligibility and improved outcomes. No studies, to our knowledge have systematically profiled comorbidities among a general HCV population. The objectives of this study were: (1) to systematically estimate the prevalence of the top 25 comorbidities in an HCV population; (2) to compare whether these most common HCV comorbidities differ between receiving antiviral treatment during the study period; and (3) to compare whether these most common HCV comorbidities differ between an HCV infected and uninfected control population.

## Methods

### Study population

Our study population was individuals within the Integrated Health Care Information System (IHCIS) National Benchmark database. IHCIS is a US medical claims database that holds claims and patient diagnosis information from over 30 health care plans in 8 census regions. Subjects for our study came from IHCIS covering claims from November 1998 to May 2006. At the time of analyses, approximately 41 million people (14%) have contributed to the database. Over 90% of patients belonging to IHCIS have medical benefits, and approximately 84% also have pharmacy benefits. The IHCIS database is de-identified and is compliant to the Health Insurance Portability and Accountability Act (HIPAA) for disclosure of protected health information.

### Disease classification systems

HCV, HCV disease severity, and comorbidities (comprising both conditions and symptoms) were initially identified using the International Classification of Disease version 9 (ICD-9 codes) in IHCIS, and then further classified according to the *Clinical Classifications Software (CCS) *2006 tool from the U.S. Agency for Healthcare Research and Quality [9]. The tool categorizes 12,000 ICD-9 codes into a manageable number of clinically meaningful categories to aid understanding patterns of diagnoses associated with particular illnesses. CCS has a single-level system that categorizes diagnoses into mutually exclusive categories, and a multi-level hierarchical system that expands the single-level categories into broader categories that provide more detail about the grouping of diagnoses; we used the latter to define comorbidities. When possible, common comorbidities were identified at the third CCS multi-level classification. However, some conditions with second level categories did not differentiate into a third-level and were reported at the second level; likewise, if first-level categories did not differentiate into a second-level, they were reported at the first-level. In addition, drug use (ICD-9 292.xx, 304.xx, 305.xx [except 305.00, 305.01-305.03], and alcohol use (291.xx, 303.xx, 305.0, 305.01-305.03, 790.3, 980.0, 980.8, 980.9, 571.x, 535.3, 357.5, 425.5, E860.0, E860.1, E860.8, E860.9) [6] were identified by ICD-9 codes since they were not defined by the CCS classification system. Multiple claims of a comorbidity were counted only once. We excluded comorbidities reflecting acute conditions, injuries/poisoning, gender-related conditions, and routine/health maintenance (Refer to Additional file 1). In order to provide greater specificity of the conditions defined within each CCS category, we cross-tabulated CCS codes with ICD-9 codes for the top 25 ranked CCS codes.

Following the convention of Feinstein [10], the term comorbidity refers to any distinct clinical entity that has existed or that may occur during the course of HCV disease. As such, these are not limited to those that are associated with HCV disease/treatment. Comorbidities associated with HCV antiviral treatment and comorbidities that exclude patients from initiating treatment were identified according to AASLD (American Association for the Study of Liver Diseases), CDC (Centers for Disease and Control and Prevention), and NIH (National Institute of Health) management practice guidelines (see Table 1).

**Table 1:** Conditions associated with HCV treatment and disease

*Conditions associated with treatment*	
Conditions associated with Interferon alfa or Ribavirin use [2] *(see *Table 3*; denoted by A)*	***Interferon only:*** neutropenia, thrombocytopenia, depression, hypothyroidism and hyperthyroidism, irritability, concentration and memory disturbances, visual disturbances, muscle aches, headaches, nausea and vomiting, low-grade fever, weight loss, insomnia, hearing loss and tinnitus, interstitial fibrosis and hair thinning ***Both Interferon and Ribavirin:*** skin irritation, fatigue ***Ribavirin only:*** hemolytic anemia, birth defects and gout
Concurrent disease for which therapy is contraindicated [2] *(See *Table 3*; denoted by B)*	major uncontrolled depressive illness; solid organ transplant (renal, heart, or lung); autoimmune hepatitis or other autoimmune condition known to be exacerbated by peginterferon and ribavirin, untreated thyroid disease; severe concurrent medical disease such as severe hypertension, heart failure, significant coronary heart disease, poorly controlled diabetes and chronic obstructive pulmonary disease

***Conditions associated with disease***	
Symptoms of HCV disease [11] *(*see Table 3*; denoted by C)*	fever, fatigue, loss of appetite, nausea, vomiting, abdominal pain, dark urine, clay-colored bowel movements, joint pain and jaundice
Extrahepatic manifestations associated with HCV infection [12] *(see *Table 3; *denoted by D)*	rheumatoid symptoms, keratoconjunctivitis sicca, lichen planus, glomerulonephritis, lymphoma, porphyria cutanea tarda, psychological disorders and essential mixed cryoglobulinemia
Conditions associated with disease progression [2,13] *(*see Table 3*; denoted by E)*	alcohol use, HIV, HBV and obesity essential hypertension [12] (evidence of reducing HCV disease progression)

### Case and control definitions

Cases were HCV patients defined as treated or untreated. Untreated cases had at least two ICD-9 diagnosis codes for chronic HCV (070.41, 070.44, 070.51, 070.54, 070.70, 070.71, 070.7, V0.262) and no α-interferon claims record, with the second code occurring 6-12 months after the first code. The second ICD-9 code was used to confirm the HCV diagnosis and its date is referred to as the HCV index date. Treated cases had at least one α-interferon claim after their first HCV ICD-9 code. If the date of the first interferon claim occurred within 6-12 months after the first HCV diagnosis code and before a second chronic HCV ICD-9 code, it was considered the index date. We were unable to capture HCV diagnoses or treatments that may have been received prior to patient enrollment in IHCIS.

Cases with multiple V0.262 codes (HCV carrier) and no other HCV codes were excluded from analyses. For study eligibility, subjects also had to have at least 24 months of follow-up in IHCIS (12 months pre- and 12 months post- the HCV index date), no gaps in health coverage, and full medical and pharmacy benefits coverage.

Controls were enrollees without HCV who were randomly selected from ICHIS, and were matched to a case (1:1) on age (within 1 year), sex, continuous medical health coverage, and enrollment period. The study period during which comorbidities were identified was the 12 months pre- and 12 months post- HCV index date. Patient age was calculated at the index date.

### Disease severity

Initially, disease severity was classified into four categories: no cirrhosis, cirrhosis (ICD-9: 571.2, 571.5, 571.6, 572.2, 572.3, 572.4), hepatocellular carcinoma (HCC; ICD-9: 155.0, 155.1, 155.2, 230.8, V10.07), and end-stage liver disease (ICD-9: 070.44, 070.41). However, no patients were identified with end-stage liver disease and 127 were diagnosed with HCC. Given the small number of patients with HCC, we grouped HCC patients with cirrhotic patients, and they were referred to as those with 'advanced liver disease'.

### Statistical analysis

The period prevalence of comorbidities during the 24-month study period was calculated. To compare the prevalence of comorbidities by treatment status for HCV patients, prevalence odds ratios (OR), 95% confidence intervals (CI), and p-values were calculated using logistic regression models, and were adjusted for age, sex, and advanced liver disease status. When comparing comorbidities in the HCV population to the matched uninfected control population, hazard ratios and 95% CI were calculated using conditional logistic regression. All analyses were performed using SAS statistical package (SAS Institute Inc, Cary, NC, Version 9.1).

## Results

### HCV study population characteristics

A total of 7411 chronic HCV patients were included in this analysis (Table 2). The study population was predominantly male (62.9%) and the median age was 49 years. Almost half of the patients received interferon during the study period (46.8%) and 19.7% had advanced liver disease. Most patients were from the northeast region of the United States (63.8%) and 83.1% of patients had HMO (health maintenance organization) or PPO (preferred provider organization) insurance. When comparing HCV infected cases vs. HCV uninfected controls, reported drug use (14.9% vs. 3.2%) and alcohol use (6.5% vs. 0.85%) was much higher among cases. Also, both drug use and alcohol use were 1.3- and 1.7- fold higher among HCV untreated patients versus the treated population.

**Table 2:** Characteristics of HCV study population

			Received HCV treatment during study period			
	**Total (n = 7411)**		**Yes (n = 3469)**		**No (n = 3942)**	
	**n**	**%**	**n**	**%**	**n**	**%**

**Gender**						
Female	2750	37.1	1134	32.7	1616	41.0
Male	4661	62.9	2335	67.3	2326	59.0
**Age category**						
0-19	18	0.2	3	0.1	15	0.4
10-19	51	0.7	18	0.5	33	0.8
20-29	142	1.9	55	1.6	87	2.2
30-39	716	9.7	342	9.9	374	9.5
40-49	3194	43.1	1583	45.6	1611	40.9
50-59	2714	36.6	1294	37.3	1420	36.0
60-64	329	4.4	137	3.9	192	4.9
65+	247	3.3	37	1.1	210	5.3
**Census Region**						
Midwest	305	4.1	142	4.1	163	4.1
Northeast	4730	63.8	2259	65.1	2471	62.7
South	700	9.4	368	10.6	332	8.4
West	369	5.0	134	3.9	235	6.0
Other^a^	1307	17.6	566	16.3	741	18.8
**Health insurance type^b^**						
Health Maintainance Organization	3247	43.8	1500	43.2	1747	44.3
Individual Plans	302	4.1	142	4.1	16.	4.1
Point of Service	755	10.2	382	11.0	373	9.5
Preferred Provider Organization	2914	39.3	1405	40.5	1509	38.3
Other	167	2.3	39	1.1	128	3.2
**Advanced liver disease**	1462	19.7	1017	29.3	445	11.3
Cirrhosis	1335	18.0	926	26.7	409	10.4
Hepatocellular carcinoma	127	1.7	91	2.6	36	0.9
**Drug use**	1101	14.9	447	12.9	654	16.6
**Alcohol use**	485	6.5	166	4.8	319	8.1

^a ^Other region includes other regions and regions which were masked for confidentiality purposes

^b ^26 patients had unknown health insurance type.

### Overall frequency of HCV comorbidities

**Figure 1:**
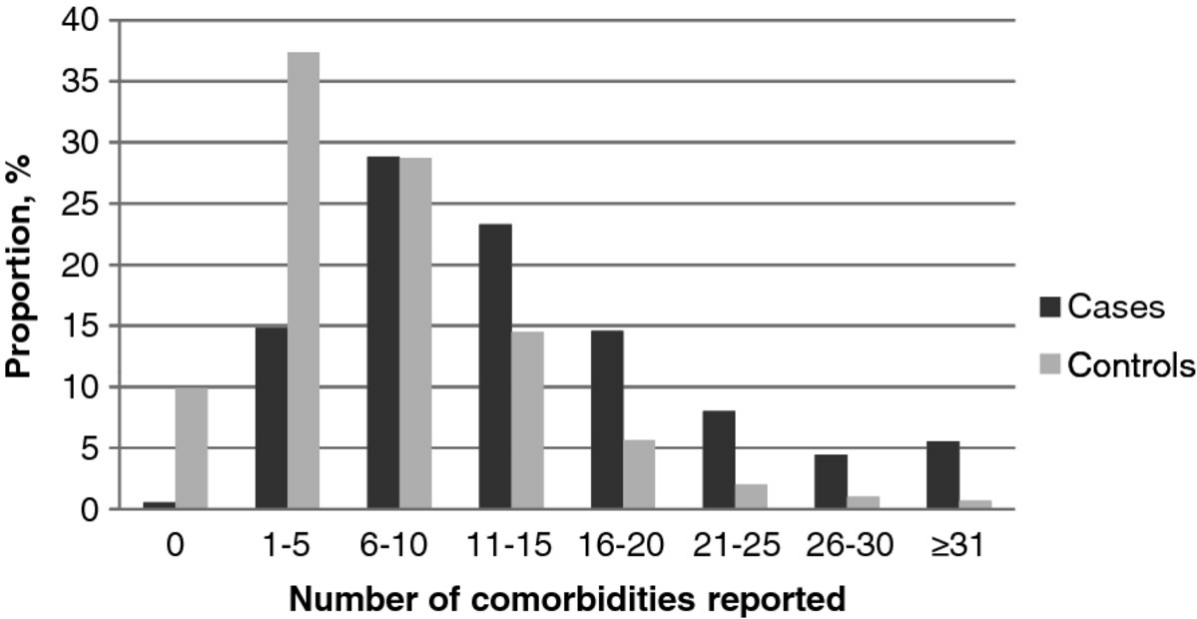
**The proportion of HCV infected cases and HCV uninfected controls reporting comorbidities**.

Comorbidities were common in this insured HCV population (Figure 1). Almost all HCV patients (99.4%) reported ≥1 comorbidity and only 41 patients (0.6%) reported none. Fifteen percent reported ≤5 comorbidities, whereas 52% reported 6-15 comorbidities, and 5.5% reported ≥31 conditions. When comparing HCV-infected patients to uninfected controls, HCV-infected patients reported almost double the number of comorbidities (101,219 vs. 53,589, respectively). More uninfected control patients (37.4%) reported ≤5 comorbidities compared to case patients (14.8%). The HCV infected population was more likely to report ≥21 comorbidities than the uninfected, 13.5% vs. 3.7%, respectively.

### Top 25 most common comorbidities

Table 3 presents a ranked list of the top 25 most common comorbidities in the overall HCV population, and also presents the frequency of these conditions among the treated and untreated HCV populations. In addition, the association (odds ratios adjusted for age, sex, and advanced liver disease) of the comorbidity between HCV treated and untreated, and between the HCV-infected (all) compared to the uninfected controls are shown. For a number of conditions where the CCS category name is denoted with "other" or was considered not to be fully explanatory, we described the most common conditions that were classified within that comorbidity by ICD-9 codes as a table footnote. Eight symptoms (denoted by category C) and eighteen comorbid conditions were identified among the top 25 list.

**Table 3:** 25 most prevalent comorbidities and symptoms in the overall HCV population stratified and compared by antiviral treatment status and control patients without HCV

Prevalence													
**Rank**	**Comorbidity**	**CCS code**	**Comorbidities** **associated with HCV treatment and disease**‡	**No. of total HCV cases (n = 7411)**	**%**	**No. of HCV treated patients (n = 3469)**	**%**	**No. of HCV untreated patients (n = 3942)**	**%**	**No. of HCV uninfected controls (n = 7411)**	**%**	Odds ratio (95%CI): treated vs untreated *	**Odds ratio (95% CI): HCV cases vs. controls**

1	Liver disease, other^a^	9.8.2	C	2779	37.5	1451	41.8	1328	33.7	221	3.0	1.34 (1.21-1.47)	19.67 (16.57-23.36)
2	Connective tissue disease, other^b^	13.8	A, D	2776	37.5	1200	34.6	1576	40.0	2050	27.7	0.81 (0.74-0.90)	1.59 (1.48-1.70)
3	Abdominal pain	17.1.7	C	2675	36.1	1212	34.9	1463	37.1	1029	13.9	0.90 (0.81-0.99)	3.55 (3.25-3.87)
4	Upper respiratory disease, and other	8.1.5	A	2640	35.6	1204	34.7	1436	36.4	2194	29.6	0.96 (0.87-1.05)	1.33 (1.24-1.43)
5	Lower respiratory disease, and other unspecified^c^	8.8.3	B°	2494	33.7	1197	34.5	1297	32.9	1332	18.0	1.06(0.96-1.17)	2.39 (2.21-2.59)
6	Essential hypertension	7.1.1	E	2416	32.6	1099	31.7	1317	33.4	1914	25.8	0.93 (0.84-1.03)	1.42 (1.32-1.53)
7	Back problems, and other^d^	13.3.3	C	2409	32.5	1046	30.2	13.63	34.6	1502	20.3	0.84 (0.76-0.92)	1.90 (1.76-2.05)
8	Non-traumatic joint disorders, other^e^	13.2.3	C	2172	29.3	877	25.3	1295	32.9	1484	20.0	0.71 (0.64-0.78)	1.66 (1.53-1.79)
9	Skin disorders, other^f^	12.4	A	2125	28.7	998	28.8	1127	28.6	1649	22.3	1.06 (0.95-1.17)	1.40 (1.30-1.51)
10	Nonspecific chest pain	7.2.5	A, C	1960	26.5	859	24.8	1101	27.9	1154	15.6	0.84 (0.76-0.94)	1.98 (1.82-2.15)
11	Disorders of lipid metabolism	3.6	C	1920	25.9	813	23.4	1107	28.1	2384	32.2	0.80 (0.72-0.89)	0.73 (0.68-0.79)
12	Malaise and fatigue	17.1.8	A	1887	25.5	933	26.9	954	24.2	866	11.7	1.18 (1.07-1.32)	2.62 (2.39-2.87)
13	Gastrointestinal disorders, other and unspecified^g^	9.12.3	C	1807	24.4	836	24.1	971	24.6	708	9.6	0.93 (0.84-1.04)	3.11 (2.81-3.43)
14	Benign neoplasm, other and unspecified^h^	2.16.2	Unk	1798	24.3	833	24.0	965	24.5	1333	18.0	1.00 (0.90-1.12)	1.48 (1.36-1.61)
15	Anemia deficiency, other	4.1.3	A	1648	22.2	983	28.3	665	16.9	441	6.0	1.99 (1.77-2.23)	4.57 (4.06-5.14)
16	Esophageal disorders^i^	9.4.1	E	1518	20.5	674	19.4	844	21.4	709	9.6	0.85 (0.76-0.96)	2.47 (2.24-2.73)
17	Upper respiratory disease, other^j^	8.9	A, B^P^	1441	19.4	650	18.7	791	20.1	1098	14.8	0.95 (0.85-1.07)	1.39 (1.27-1.51)
18	Allergic reactions^k^	17.1.9	A	1104	14.9	616	17.8	488	12.4	714	9.6	1.60 (1.41-1.83)	1.64 (1.48-1.81)
19	Genitourinary symptoms and ill-defined conditions^l^	10.1.8	Unk	1098	14.8	483	13.9	615	15.6	677	9.1	0.89 (0.78-1.01)	1.73 (1.56-1.92)
20	Substance-related mental disorders	5.2.2	E	1091	14.7	443	12.8	648	16.4	818	3.2	1.07 (0.94-1.22	1.39 (1.26-1.53)
21	Blindness and vision defects	6.7.4	A	1078	14.6	512	14.8	566	14.4	818	11.0	1.07 (0.94-1.22)	1.80 (1.62-2.01)
22	Depressive disorder	5.9.2	A, B, D	1028	13.9	489	14.1	539	13.7	312	4.2	1.06(0.92-1.21)	3.72 (3.25-4.27)
23	Diabetes mellitus without complication	3.2	B	1025	13.8	426	12.3	599	15.2	627	8.5	0.74 (0.65-0.85)	1.80 (1.62-2.01)
24	Viral infections, other^m^	1.3.3	Unk	1019	13.8	447	12.9	572	14.5	663	8.9	0.87 (0.76-0.99)	1.63 (1.47-1.81)
25	Eye disorders, other^n^	6.7.6	A	1001	13.5	499	14.4	502	12.7	758	10.2	1.23 (1.08-1.41)	1.38 (1.25-1.53)

*CCS *Clinical Classification Code

*Odds ratios are adjusted for age, sex, and advanced liver disease status

‡ Comorbidities associated with *HCV treatment: *(A) conditions associated with inteferon alfa or ribavirin use and (B) concurrent disease for which therapy is contraindicated; and *HCV disease: *(C) symptoms of HCV disease, (D) extrahepatic manifestations associated with HCV infection, and (E) conditions associated with disease progression; Unk, unknown

^a ^Of the patients who had other liver diseases, they reported abnormal liver function (69.5%), elevated transaminase/lactate dehydrogenase (26.7%), abnormal serum enzyme levels (13.8%), ascites (10.7%), and hepatomegaly (9.0%)

^b ^Of the patients who had other connective tissue disease, they mainly reported pain in limb (38.7%), myalgia and myositis unspecified (18.8%), swelling of limb (12.5%), and spasm of muscle (9.7%)

^c ^Of the patients who had other and unspecified lower respiratory disease, they mainly reported coughs (48.5%), other respiratory abnormalities (35.6%), shortness of breath (34.1%), and other lung disease

^e ^Of the patients who had other non-traumatic joint disorders, they mainly reported joint pain in the leg (25.5%), shoulder (24.1%), ankle (15.9%), and pelvis (14.4%)

^f ^Of the patients who had other skin disorders, they mainly reported other nonspecified skin eruptions (22.3%), sebaceous cyst (14.3%), actinic keratosis (12.3%), and other seborrheic keratosis (10.8%)

^g ^Of the patients who had other unspecified gastrointestinal disorders, they mainly reported diarrhea (26.7%), splenomegaly (20.3%), irritable bowel syndrome (11.9%), and unspecified site of abdominal/pelvic swelling (10.6%)

^h ^Of the patients who had other unspecified benign neoplasm, they mainly reported benign neoplasm of the large bowel (39.2%), skin trunk (14.3%), and unspecified skin (11.5%)

^i ^Of the patients who had esophageal disorders, they mainly reported esophageal reflux (62.6%), reflux esophagitis (21.1%), esophagitis unsepcified (14.5%), and esophageal varices without bleeding (13.7%)

^j ^Of the patients who had other upper respiratory disease, they mainly reported unspecified allergic rhinitis (43.0%), rhinitis due to pollen (13.6%), chronic rhinitis (12.4%), nasal cavity/other sinus disorder (12.2%), epistaxis (11.1%), and deviated nasal septum (11.0%)

^k ^Of the patients who had allergic reactions, they mainly reported unspecified dermatitis (65.9%) and other atopic dermatitis (11.1%)

^l ^Of the patients who had genitourinary symptoms and ill-defined conditions, they mainly reported hematuria (32.0%), urinary frequency (20.1%), and dysuria (18.9%)

^m ^Of the patients who had other viral infections, they mainly reported unspecified viral infections (42.8%), unspecified viral warts (22.0%), and unspecified herpes simplex (11.4%)

^n ^Of the patients who had other eye disorders, they mainly reported unspecified tear film insufficiency (19.7%), other vitreous opacities (15.9%), and vitreous degeneration (9.5%)

^o ^Chronic obstructive pulmonary disease (COPD) may be misclassified under lower respiratory disease which is a antiviral treatment contraindication [14]

^p ^Upper respiratory disease, comprising mainly of rhinitis, may reflect an under-diagnosis of thyorid disorders, which is known to be associated with HCV [15].

Among the top ranked conditions, the majority of the comorbidities were grouped into 7 wider body system classes as follows: *diseases of the digestive system *(liver diseases, other [rank 1], gastrointestinal disorders, other and unspecified [rank 13], esophageal disorders [rank 16]), *disease of the musculoskeletal system and connective tissue *(connective tissue disease, other [rank 2], back problems, other [rank 7], non-traumatic joint disorders, other [rank 8]), *diseases of the respiratory system *(upper respiratory infections, other [rank 4], unspecified lower respiratory disease, other [rank 5], upper respiratory disease, other [rank 17]), *diseases of the circulatory system *(essential hypertension [rank 6], non-specific chest pain [rank 10]), *endocrine, nutritional and metabolic diseases and immunity disorders *(disorders of lipid metabolism [rank 11] and diabetes mellitus without complication [rank 23]), *mental disorders *(alcohol and substance-related mental disorders [rank 20] and depressive disorders [rank 22]), and *diseases of the nervous systems and sense organs *(blindness and vision defects [rank 21], eye disorders, other [rank 25]). *Symptoms and signs influencing health status *(malaise and fatigue [rank 12] and allergic reactions [rank 18]) were also common in this HCV population.

Other liver diseases (37.5%) was the most common comorbidity (inclusive of symptoms) identified in this HCV population, and of these patients, 70% reported abnormal liver function, 26.7% elevated aminotransaminases, 13.8% abnormal serum enzyme levels, 10.7% ascites, and 9.0% hepatomegaly. In addition, these patients were more likely to have been treated during the study period (odds ratio, OR = 1.34, 95% CI: 1.21-1.47).

### Comorbidities associated with HCV antiviral treatment

We note that several conditions associated with antiviral treatment (see Table 3; A) were ranked among the top 25 comorbidities: muscle aches (denoted within connective tissues disease, 34.6% of those treated), sinusitis (within upper respiratory infections, 34.7%), skin irritation (within skin disorders, 28.8% and allergic reactions, which comprised mainly unspecified dermatitis, 17.8%), malaise and fatigue (26.9%), anemia (28.3%), visual disturbances (within blindness and vision defects, 14.8% and eye disorders, other, 14.4%) and depressive disorders (14.1%), however, only those reporting malaise and fatigue (OR = 1.18, 95% CI: 1.07-1.32), anemia deficiency (OR = 1.99, 95% CI: 1.77-2.23) and eye disorders, other (OR = 1.23, 95% CI: 1.08-1.41) were significantly more likely to be treated during the study period. Thyroid disorders, which are contraindicated for treatment, did not rank in the top 25 comorbid conditions (prevalence, 12.5%). However, upper respiratory disease (19.4%; rank 17), was comprised mainly of rhinintis, which is known to be associated with thyroid disorders [16]. Therefore, the high prevalence of rhinitis may suggest the under-diagnosis of thyroid disorders in this HCV population. There was no difference in treatment status for patients with upper respiratory disease, although patients with thyroid disorders were more likely to be treated (OR = 1.60; 95% CI: 1.39-1.85) in our study population (data not shown).

Several conditions which preclude patients from treatment (see Table 3; B) were also among the top comorbidities; diabetes mellitus without complications (13.8%), and depressive disorders (13.9%), however only those with diabetes mellitus without complications were less likely to be treated during the study period (OR = 0.74, 95% CI: 0.65-0.85). Non-specific chest pain (26.5%) could be a symptom of HCV disease or result as an adverse event from pegylated interferon treatment (mainly Pegasys [17]), and it was identified in our ranked list of comorbidities. Although we cannot determine which condition chest pain is associated with, these patients were less likely to be treated (OR = 0.84; 95% CI: 0.76-0.94). We did not identify chronic obstructive pulmonary disease (COPD) in our top list of comorbidities, an antiviral treatment contraindication, however, it ranked only slightly lower than our top 25 (prevalence, 13%).

Although disorders of lipid metabolism [rank 11] was not defined by HCV management guidelines in Table 1, HCV infection is known to be associated with enhanced lipogenesis, reduced secretion, and β-oxidation of lipids; therefore, it was not surprising that it ranked in our most frequent list of comorbidities [18].

### Comorbidites associated with HCV disease

We also found that several conditions associated with HCV disease ranked among the top 25 comorbidities. Among the symptoms associated with HCV disease (see Table 3; C), joint pain (including back problems (32.5%) and joint disorders (29.3%)) and abdominal pain (36.1%) were identified; and these patients were less likely to be treated. In addition, gastrointestinal disorders (24.4%) comprising mainly of diarrhea, splenomegaly, irritable bowel syndrome and unspecified site of abdominal/pelvic swelling may also result in abdominal pain.

Among the common extrahepatic manifestations seen among chronic HCV patients (see Table 3; D), we identified rheumatoid symptoms (denoted within connective tissue disease and malaise/fatigue) and psychological disorders (within substance-related mental disorders and depressive disorders) to be the most prevalent in our study population.

Among the comorbidities known to be associated with liver disease progression (see Table 3; E), substance-related mental disorders, (associated with drug and alcohol use) and essential hypertension ranked in our top list, and those with substance-related mental disorders were less likely to be treated (OR = 0.70, 95% CI: 0.61-0.80). Although HIV, HBV, obesity did not rank in our list, the prevalence of these comorbidities were 3.2%, 8.4%, and 3.8%, respectively. Approximately 20% of the HCV population had advanced liver disease (cirrhosis and HCC); and these patients are at risk of developing further complications from cirrhosis which occur as a result of portal hypertension and hyperdynamic circulation [19]. This is evidenced by the high ranking of esophageal disorders (particularly esophageal varices) in our ranking of most common comorbidities.

### Comorbidities not known to be associated with HCV antiviral treatment or disease

Of the top 25 ranked list of comorbidities, the conditions (n = 3) that were not known to be associated with antiviral treatment or disease, and hence were least expected, were benign neoplasms, upper respiratory disease, genitourinary symptoms and ill-defined conditions, and viral infections (which was defined mainly by unspecified viral infections, viral warts, and unspecified herpes simplex). These conditions were about 1.3- to 1.7-fold higher in the HCV-population compared to the HCV uninfected controls.

### Ranking of top 25 comorbidities according to HCV status

The top ranking comorbidities among the overall HCV population were consistently identified in the top 25 comorbidities list of stratified groups of HCV treated, untreated, and controls populations, however with different prevalence and ranking orders (data not shown). There were a few exceptions. Specifically, diabetes mellitus without complication did not rank among the top-ranked list of comorbidities among HCV treated patients, whereas patients with thyroid disorders (13.4%; mainly hypothyroidism) were recorded. Among the HCV untreated patients, eye disorders and allergic reactions were not seen, whereas cardiac dysrhythmias (14.0%) and osteoarthritis (13.5%) ranked. Amongst the uninfected control group, other liver diseases, anemia deficiency, substance-related mental disorders, and depressive disorders were not present in the top 25 list, whereas bone disease and musculoskeletal deformities, other (9.1%), ear and sense organ disorders, other (8.8%), non-malignant breast conditions (8.7%), and cardiac dysrhythmias (8.3%) ranked.

Comorbidities in the HCV population (regardless of treatment status) were more prevalent than in the controls, except for disorders of lipid metabolism (32.2% among controls vs. 25.9% among cases; OR = 0.73; 95% CI: 0.68-0.79). Not surprising, HCV cases with other liver diseases (OR = 19.67; 95% CI: 16.57-23.36) had the highest OR compared to the HCV controls, followed by those with anemia deficiency (OR = 4.57; 95% CI: 4.06-5.14) and depressive disorder (OR = 3.72; 95% CI: 3.25-4.27).

## Discussion

To our knowledge, this is the first study to systematically profile non-selected comorbidities in an HCV population. Nearly every HCV patient in our study had at least one comorbidity, and in general, these comorbidities were more common in the overall HCV infected case patients compared to the uninfected controls. In addition, comorbidities covered a wide range of diseases and symptoms affecting a number of body systems. Our findings substantiate that HCV is a systemic disease with many extra-hepatic features which highlight the urgency to treat these extra-hepatic conditions regardless of the liver disease itself. Individualized anti-viral treatment decisions for HCV infection should weigh the potential risks and benefits of both hepatic and extra-hepatic manifestations to increase the likelihood of treatment success.

Our insured study population reported a 47% current treatment rate, which is comparable to treatment rates (10-51%) in other US medical claims database studies [20-22]. There are multiple reasons why HCV patients may not be treated which include disease stage, patient choice and lack of access to liver specialists [23]. Non-liver specialists may decide that the patient is ineligible for treatment based on a list of contraindications, whereas liver specialists may otherwise safely treat the patient with relative contraindications (i.e. patients with cirrhosis and moderate thrombocytopenia) since most treatment regimens are individualized to achieve maximal sustained virological response. Treatment rates appear to be declining in the US, and it is estimated that from 2002-2030, only 14.5% of liver-related deaths caused by HCV will be prevented [24]; therefore, it is urgent to understand what barriers may exist that are preventing treatment uptake as the burden of HCV morbidity and mortality increases.

Up to one-third of our HCV population had a comorbidity that could potentially make them ineligible for antiviral HCV treatment. This indicates that a high proportion of the HCV population could potentially receive effective treatment if their comorbid conditions could be managed. A recent review has reported that the benefits of treatment can potentially reach a wider HCV population by including targeted groups such as active drug users and persons those with psychiatric illnesses [25]. These patients were able to complete antiviral therapy, achieve a sustained virological response, and did not develop adverse events that would require discontinuation of treatment. However, it was recommended that individualized management of these patients could be integrated within addiction and treatment services [26]. These studies suggest that some comorbidities can be effectively managed, increasing access to HCV treatment with successful outcomes.

Our findings confirm previous studies as we consistently observed the known comorbidities associated with HCV antiviral treatment and disease among our top ranked list, highlighting that our systematic evaluation supports how common these conditions are among the HCV infected population. However, three conditions, benign neoplasms, genitourinary symptoms, and viral infections, were identified in our ranked list and have not been previously reported to be associated with treatment or disease progression. The prevalence of benign neoplasms were mainly of the large bowel and skin (trunk and unspecified sites), which could possibly be explained by metastasis to these sites among HCV patients with HCC but its pathophysiology is unclear and warrants further study [27,15]. In our study, the prevalence of HCC was low, but we cannot exclude the possibility that this may be an underestimation if patients were diagnosed prior to the study period. HCV patients with genitourinary symptoms were mainly of hematuria and proteinuria [28] which could be clinical manifestations of glomerular kidney disease that is known to be associated with cryoglobulinemia and membranoproliferative glomerulonephritis [29]. For the unexpected finding of viral infections, the significance is difficult to interpret given the lack of specificity as almost half the cases were coded as non-specific viral infections.

Our HCV infected population was more likely to report comorbidities than the uninfected controls. This may be explained in part by the higher-risk or unhealthy behavior (e.g., alcohol and drug users) of those infected with HCV, making them more vulnerable to acquire new morbidities than the uninfected population. Indeed, prevalence of alcohol use and drug-use were much higher among the HCV infected compared to the uninfected population (alcohol; 7% vs. < 1%; drug use 15% vs. 3%, respectively). In addition, HCV-infected patients with health insurance may have closer follow-up care and higher rates of screening for other diseases than the uninfected, hence increasing the reporting of conditions. HCV-infected patients may also have more comorbidities because of the illness itself, which may place them at higher risk for other conditions. However, we identified disorders of lipid metabolism to be significantly lower among the HCV population compared to the uninfected population. This finding is not clearly understood since HCV infection is associated with enhanced lipogenesis that may lead to the pathological development of steatosis and metabolic syndromes such as insulin resistance, obesity, and HCC [18].

Our study has several limitations. Firstly, as we used a medical claims database, we did not have liver histology data to confirm stage of disease and thus cannot exclude the possibility of case ascertainment bias as the selection of cases required at least two clinical ICD-9 HCV diagnosis codes for (e.g. patients with more advanced liver disease or on anti-viral treatment would have more clinical visits). Secondly, since data were limited on HCV genotypes (< 1%) and unavailable for racial groups, we were unable to match on these factors in our analyses although HCV disease/treatment efficacy is known to vary by genotype and race [30]. Thirdly, we relied on clinical coding for disease stage so the potential for misclassification of HCV diagnosis and liver disease stage cannot be ruled out. In addition, we cannot exclude the possibility of inconsistent coding or misclassification of comorbidities in our study; for example, COPD, which did not rank in our top 25 list, could have been misclassified under lower respiratory disease which ranked 5. Similarly, cutaneous conditions such as lichen planus and porphyria cutanea tarda which are commonly seen extrahepatic manifestations did not rank in the top 25, however, it is possible that these dermatologic conditions could be misclassified under allergic reactions [rank 18], which was mainly composed of unspecified dermatitis (65.9% of HCV patients with allergic reactions). Furthermore, given the cross-sectional design of this study, we were unable to establish the temporal sequence of events, i.e. whether a comorbidity, such as anemia deficiency, allergic reactions or eye disorders occurred prior to treatment, during treatment, or were treatment side effects. Lastly, patients captured in a medical claims database may be a biased selection of the HCV population as it does not reflect the population who do not have access to healthcare, so estimates of these comorbidities should be extrapolated with caution to the general HCV population. However, our results could be generalized to the privately insured population. According to population-based data from the National Health and Nutrition Examination Survey (NHANES), 61% of those chronically infected with HCV had health insurance and among those insured, half of them had private health insurance [31]. Despite these limitations, our study includes a much broader HCV population than previous studies, which were limited to select populations of US veterans [14,32,33], dialysis patients [34], psychiatric patients [35] and drug users [36]; and represents an insured population that would have access to treatment if comorbidities were able to be managed.

## Conclusions

In summary, this study identified a wide range of highly prevalent comorbidities, which may have implications for HCV prognosis, management and treatment success. With over 4 million Americans currently infected with HCV, it is one of the most pressing public health challenges. Effective strategies to manage and control comorbidities among chronic HCV patients may result in increased access to treatment, thereby reducing the future burden of HCV disease and its manifestations.

## Competing interests

Karly S Louie undertook this study as part of a Pre-doctoral Fellowship within Worldwide Epidemiology of GlaxoSmithKline. Samantha St. Laurent, Ulla M Forssen, Linda M Mundy and Jeanne M Pimenta are full-time employees of GlaxoSmithKline.

## Authors' contributions

KSL: Conception and design of the study; generation, collection, assembly, analysis and interpretation of data; drafting and revision of the manuscript; approval of the final version of the manuscript. SSL: Generation, collection, assembly, analysis of data; revision of the manuscript; and approval of the final version of the manuscript. UMF: Revision of the manuscript and approval of the final version of the manuscript. LMM: Revision of the manuscript and approval of the final version of the manuscript. JMP: Conception and design of the study; interpretation of the manuscript; drafting and revision of the manuscript; and approval of the final version of the manuscript

## Pre-publication history

The pre-publication history for this paper can be accessed here:

http://www.biomedcentral.com/1471-2334/12/86/prepub

## Supplementary Material

Additional file 1 **Appendix A**. Excluded comorbidities that reflect acute conditions, injuries/poisoning, gender-related conditions, and routine/health maintenance. Click here for file
