# Supplementary material for: The high comorbidity burden of the hepatitis C virus infected population in the United States
Source: BMC Infect Dis. 2012 Apr 11;12:86. doi: 10.1186/1471-2334-12-86 (PMC3342214; doi:10.1186/1471-2334-12-86)
Supplement: Additional file 1 — Appendix A. Excluded comorbidities that reflect acute conditions, injuries/poisoning, gender-related conditions, and routine/health maintenance. [file 1471-2334-12-86-S1.PDF]

Appendix A: Excluded comorbidities that reflect acute conditions, injuries/poisoning, gender-related conditions, and routine/health maintenance

CCS Code	Comorbidity	Reason for exclusion<br><br>1=Acute conditions; 2=injuries/poisoning; 3=gender-related conditions; 4=routine/health maintenance; 5=unspecified
1.1.2	Septicemia (except in labor)	1
1.1.4	Other bacterial infections	1
1.1.3	Sexually transmitted infections (not HIV or hepatitis)	1
1,5	Immunizations and screening for infectious disease	4
2.5	Cancer of breast	3
2.6.1	Cancer of uterus	3
2.6.2	Cancer of cervix	3
2.7.1	Cancer of ovary	3
2.7.2	Cancer of other female genital organs	3
2.8.1	Cancer of prostate	3
2.8.2	Cancer of testis	3
2.8.3	Cancer of other male genital organs	3
2.16.1	Benign neoplasm of uterus	3
4.1.1	Acute posthemorrhagic anemia	1
8.1.2	Influenza	1
8.1.4	Acute bronchitis	1
10.3.3	Endometriosis [	3
10.3.4	Prolapse of female genital organs	3
10.3.5	Menstrual disorders	3
10.3.6	Ovarian cyst	3
10.3.7	Menopausal disorders	3

10.3.8	Female infertility	3
10.3.9	Other female genital disorders	3
11.1.1	Sterilization	3
11.1.2	Other contraceptive and procreation management	3
11.2.1	Spontaneous abortion	3
11.2.2	Induced abortion	3
11.2.3	Postabortion complications	3
11.3.1	Ectopic pregnancy	3
11.3.7	Other complications of pregnancy	3
11.3.2	Hemorrhage during pregnancy	3
11.3.3	Hypertension complicating pregnancy	3
11.3.4	Early or threatened labor	3
11.3.5	Prolonged pregnancy	3
11.3.6	Diabetes or abnormal glucose tolerance complication	3
11.4.1	Malposition; malpresentation	3
11.4.2	Fetopelvic disproportion; obstruction	3
11.4.3	Previous cesarean section	3
11.4.4	Fetal distress and abnormal forces of labor	3
11.4.5	Polyhydramnios and other problems of amniotic cavity	3
11.5.1	Umbilical cord complication	3
11.5.2	Trauma to perineum and vulva	3
11.5.3	Forceps delivery	3
11.6.1	Postpartum hemorrhage	3
11.6.2	Complications of the puerperium	3
11.6.3	Cervical incompetence	3
11.6.4	Rhesus isoimmunization	3
11.6.5	Intrauterine death	3

11.6.6	Failed induction	3
11.6.7	Other obstetrical trauma	3
11.6.8	Other and unspecified complications of birth	3
11.7.1	Normal delivery	3
11.7.2	Multiple gestation	3
11.7.3	Outcome of delivery	3
15.1	Liveborn	3
15.2	Short gestation; low birth weight; and fetal growth	3
15.3	Intrauterine hypoxia and birth asphyxia	3
15.4	Respiratory distress syndrome	3
15.5	Hemolytic jaundice and perinatal jaundice	3
15.6	Birth trauma	3
15.7.1	Respiratory conditions of fetus and newborn; other	3
15.7.2	Infections specific to the perinatal period	3
15.7.3	Endocrine and metabolic disturbances of fetus and	3
15.7.4	Other and unspecified perinatal conditions	3
15.7	Other perinatal conditions	1
16.7	Sprains and strains	2
16.4.1	Concussion	2
16.4.2	Other intracranial injury	2
16.5	Crushing injury or internal injury	2
16.8	Superficial injury; contusion	2
16.9	Burns	2
16.11.1	Poisoning by psychotropic agents	2
16.11.2	Poisoning by other medications and drugs [	2
16.11.3	Poisoning by nonmedicinal substances	2

16.12	Other injuries and conditions due to external causes	2
16.1	Joint disorders and dislocations; trauma-related	2
16.10.1	Complication of device; implant or graft	2
16.10.2	Complications of surgical procedures	2
16.12	Other injuries and conditions due to external causes	2
17.2.1	Rehabilitation care; fitting of prostheses	4
17.2.2	Administrative/social admission	4
17.2.3	Medical examination/evaluation	4
17.2.4	Other aftercare	4
17.2.5	Other screening for suspected conditions	5
259	Unclassified	5
2601	E Codes: Cut/pier	2
2602	E Codes: Drowning	2
2603	E Codes: Fall	2
2604	E Codes: Fire/burn	2
2605	E Codes: Firearm	2
2606	E Codes: Machinery	2
2607	E Codes: Motor vehicle	2
2608	E Codes: Pedal cy	2
2609	E Codes: Pedestrian	2
2610	E Codes: Transportation	2
2611	E Codes: Natural/	2
2612	E Codes: Overexertion	2
2613	E Codes: Poisoning	2
2614	E Codes: Struck b	2
2615	E Codes: Suffocat	2

2616	E Codes: Adverse	2
2617	E Codes: Adverse	2
2618	E Codes: Other specified	2
2619	E Codes: Other specified	2
2620	E Codes: Unspecified	2
2621	E Codes: Place of	2
